# An experience of liver transplantation in Latin America: a medical center in Colombia

**Published:** 2015-03-30

**Authors:** Oscar Santos, Mauricio Londoño, Juan Marín, Octavio Muñoz, Álvaro Mena, Carlos Guzmán, Sergio Hoyos, Juan Restrepo, María Arbeláez, Gonzalo Correa

**Affiliations:** 1 Hepatology and Liver Transplantation Unit, Pablo Tobon Uribe Hospital, Medellin, Colombia; 2 Gastrohepatology Research Group, University of Antioquia, Medellin, Colombia; 3 Epidemiology Research Group, National Faculty of Public Health, University of Antioquia, Medellin, Colombia

**Keywords:** liver transplantation, cirrhosis, liver graft, rejection, immunosuppressive

## Abstract

**Objectives:**

Liver transplantation is the treatment of choice for acute and chronic liver failure, for selected cases of tumors, and for conditions resulting from errors in metabolism. This paper reports the experience of a medical center in Latin America.

**Methods:**

Were conducted 305 orthotopic liver transplantations on 284 patients between 2004 and 2010. Of these patients, 241 were adults undergoing their first transplantation.

**Results:**

The average age of patients was 52 years old, and 62% of the individuals were male. The most common indication was alcoholic cirrhosis. The rate of patient survival after 1 and 5 years was 82 and 72% respectively. The rate of liver graft survival after 1 and 5 years was 78 and 68% respectively. The main cause of death was sepsis. Complications in the hepatic artery were documented for 5% of the patients. Additionally, 14.5% of the patients had complications in the biliary tract. Infections were found in 41% of the individuals. Acute rejection was observed in 30% of the subjects, and chronic rejection in 3%.

**Conclusion:**

In conclusion, liver transplantation at our medical center in Colombia offers good mid-term results, with a complication rate similar to that reported by other centers around the world.

## Abstract (es)

**Objetivos:**

El trasplante hepático es el tratamiento de elección para la falla hepática aguda y crónica, casos seleccionados de tumores y enfermedades por errores en el metabolismo. En este documento se reporta la experiencia de un centro en Latinoamérica.

**Métodos:**

Entre el 2004 y el 2010 se realizaron 305 trasplantes ortotópicos de hígado en 284 pacientes. De ellos, 241 corresponde a primer trasplante en adultos.

**Resultados:**

La edad promedio fue 52 años y el 62% eran hombres. La principal indicación fue cirrosis por alcohol. La supervivencia de los pacientes a 1 y 5 años fue de 82 y 72% respectivamente y del injerto hepático a 1 y 5 años fue de 78 y 68% respectivamente. La principal causa de muerte fue sepsis. Se documentaron complicaciones en la arteria hepática en el 5% y complicaciones en la vía biliar en el 14.5%. Se encontraron infecciones en el 41% de los pacientes. Rechazo agudo se presento en el 30% y rechazo crónico en el 3%.

**Conclusión:**

El trasplante hepático en nuestro centro en Colombia ofrece buenos resultados a mediano plazo, con una tasa de complicaciones similar a lo reportado en otros centros del mundo.

## Introduction

Liver transplantation (LT) is currently considered to be the treatment of choice for chronic liver failure, acute liver failure with indicators of poor prognosis, appropriately selected primary liver tumors, and some conditions resulting from errors in metabolism 1. Great advances have taken place since 1963, when Dr. Thomas Starzl performed the first LT on a human patient 2. At first, results were disappointing; however, they are now much better due to the fact that surgical techniques have been refined, perioperative care has improved, the immune suppressants used are more effective, and patients are selected more appropriately. A recent study conducted by Thuluvath on the long-term results obtained by American medical centers (OPTN/SRTR information) found that patient survival rates after 1, 5, and 10 years are 88.4, 73.8, and 60% respectively 3. Despite these favorable results, LT patients may experience serious complications that may even lead to death. Such complications include infections and vascular and biliary complications 4. In Colombia more than 1,700 liver transplantations were performed between the late 1990's and 2010. However, aside from a report of an early experience 5, there is currently no reliable information available regarding the rates of patient survival and complications. We therefore decided to report the experiences of the Liver Transplantation Research Group at the Pablo Tobon Uribe Hospital, in Medellin, Colombia. 

## Materials and Methods

Between February 2004 and December 2010, 305 orthotopic liver transplantations were performed on 272 adults and 33 children at the Pablo Tobon Uribe Hospital in Medellin, Colombia. All donors were deceased. This is a retrospective descriptive study included all patients with liver transplantation adults and were excluded pediatric patients, retransplantation and multivisceral transplants. Of the adult patients, 241 were undergoing their first liver transplantation. Demographic, morbidity, and mortality data were obtained through a retrospective review of medical records and the liver transplantation database. Our multidisciplinary team of adult liver transplant is constituted by 3 transplant surgeons, 4 hepatologists, 4 anesthesiologists, 1 psychologist, 1 social worker and a nurse coordinator. This procedure was authorized by the Hospital's ethical review board. A staging of liver disease severity was carried out for all patients using the Child-Turcotte-Pugh (Child) classification and the MELD score. All patients with hepatocellular carcinoma (HCC) had to comply with the Milan criteria in order to undergo transplantation. Individuals with acute liver failure underwent transplantation when they complied with the King's College criteria for poor prognosis. The conventional immunosuppressive treatment consisted of cyclosporine or tacrolimus, azathioprine or mycophenolate mofetil, and steroids. The latter were suspended 3 to 6 months after the procedure. We aimed to discontinue azathioprine or mycophenolate mofetil after the first year in all cases, except for those patients with autoimmune liver disease. Individuals with immunoglobulin G for cytomegalovirus (CMV) positive donor/negative receptor were treated with ganciclovir/valganciclovir prophylaxis for the first three months. The patients who had two recognized risk factors for invasive fungal infections received Fluconazole for seven days. Additionally, all individuals received Aciclovir for 30 days and Trimethoprim/Sulfamethoxazole for six months. Patients with chronic hepatitis B infection continued to be treated with oral antiviral therapy, and intramuscular immunoglobulin against hepatitis B was added for an indefinite period. In the event of moderate to severe acute rejection confirmed through biopsy, treatment with methylprednisolone bolus was used. Some patients with renal dysfunction who were not candidates for a combined liver and kidney transplantation, fell into the B or C category of the Child classification, and had ascites were administered Basiliximab in order to allow for a later introduction of the calcineurin inhibitor. The surgical technique used in the vena cava was Piggy-back, with no need for a veno-venous bypass in any patient. The conventional biliary anastomosis was choledocho-choledochostomy without the use of a T-tube and with hepaticojejunostomy according to the transplant surgeon's judgment. All patients were transferred to the intensive care unit (ICU) in the postoperative period, and an early extubation protocol was implemented. 

The statistical analysis carried out was based on descriptions of various socio-demographic and clinical variables amongst the patients studied. These variables included: pre-transplant conditions, the etiology of the liver disease, condition severity as classified by the Child and MELD instruments, intraoperative variables, and postoperative variables such as complications, ICU and hospital stay, and graft and patient survival. First, the variables' distribution type was determined, and a bivariate analysis was conducted using the x^2^ test for the categorical variables and the Mann-Whitney U non-parametric test to compare ranges between independent groups. An analysis of survival was carried out, using the Kaplan Meier curve to analyze the "loss of graft" and "patient death" outcomes after 1 and 5 years (in both cases).

## Results

### General data

**Figure 1.**
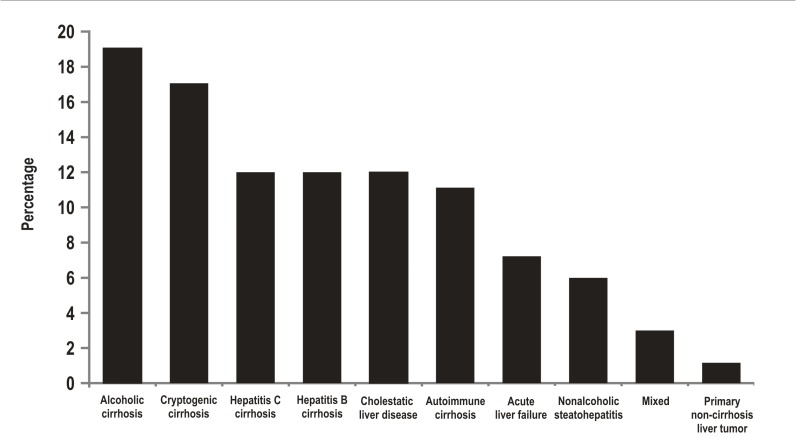
Aetiology of liver disease in liver transplant patients, Pablo Tobón Uribe Hospital, Medellín, Colombia, n: 241 patients.

The average recipient age was 52 years old (15 to 72 years old) and 62% of the patients were male. Additionally, 50% of the patients were Child C classified. The average MELD score was 19. The etiology of the liver disease is described in Figure 1. Also, 80% of the transplant patients had cirrhosis, the main cause being liver disease due to alcohol. The indication for liver transplantation was HCC for 15% of the cases, and acute liver failure for 7% of cases. The cases labeled as "mixed etiology" included patients with hemochromatosis, Wilson's disease, polycystic liver disease, amyloidosis, and Budd Chiari syndrome. In addition, two patients with primary liver tumors and no cirrhosis received a transplant. One of those patients had fibrolamellar HCC and the other had epithelioid hemangioendothelioma. Donor characteristics as well as surgical and hospital stay times are shown in Table 1. The average age of the donors was 35 years old. The cause of death was traumatic brain injury for 60% of the donors and stroke for 30% of them. The average time spent on the waitlist was 32 days. 

**Table 1.**
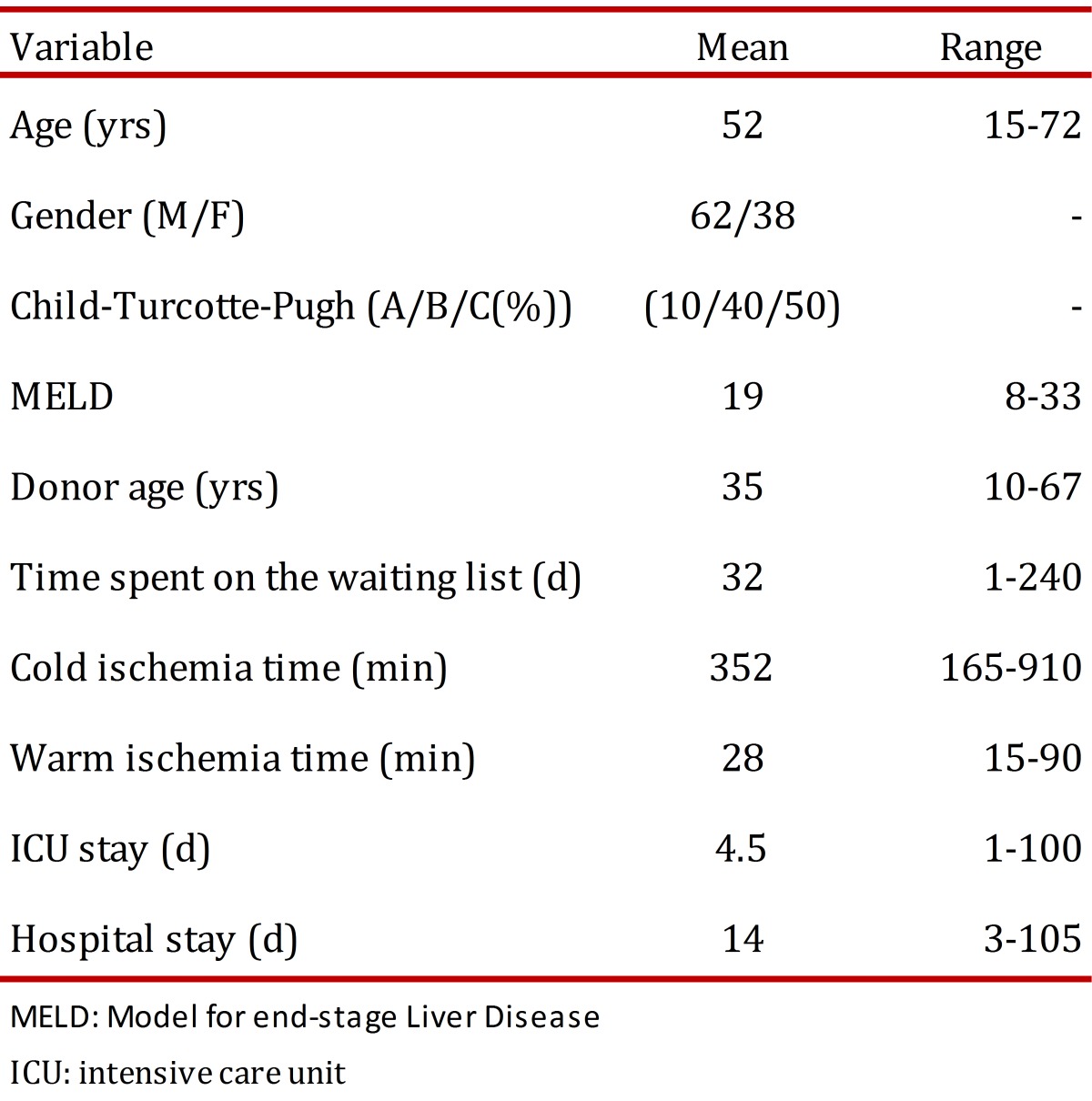
Clinical characteristics of donors and recipientsof liver transplants, Pablo Tobón Uribe Hospital, Medellín, n: 241 patients.

### Survival

**Figure 2.**
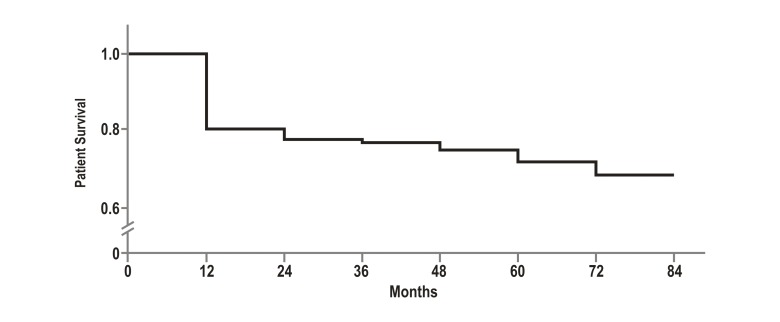
Survival rates of liver transplant patients, Pablo Tobón Uribe Hospital, Medellín, Colombia, n: 241 patients.

The rate of patient survival was 82% after 1 year and 72% after 5 years (Fig. 2). The graft survival rate was 78% after 1 year and 68% after 5 years. The leading cause of death was sepsis. Additional causes are shown in Table 2. Similarly, 40% of deaths occurred early (during the first 3 months); their causes were sepsis, massive bleeding and primary graft dysfunction. All the deaths due to massive bleeding occurred within the first two years after joining the liver transplantation program, and there were no deaths due to this cause during subsequent years. Late causes of death included cardio-cerebro-vascular disease and kidney failure. 

**Table 2.**
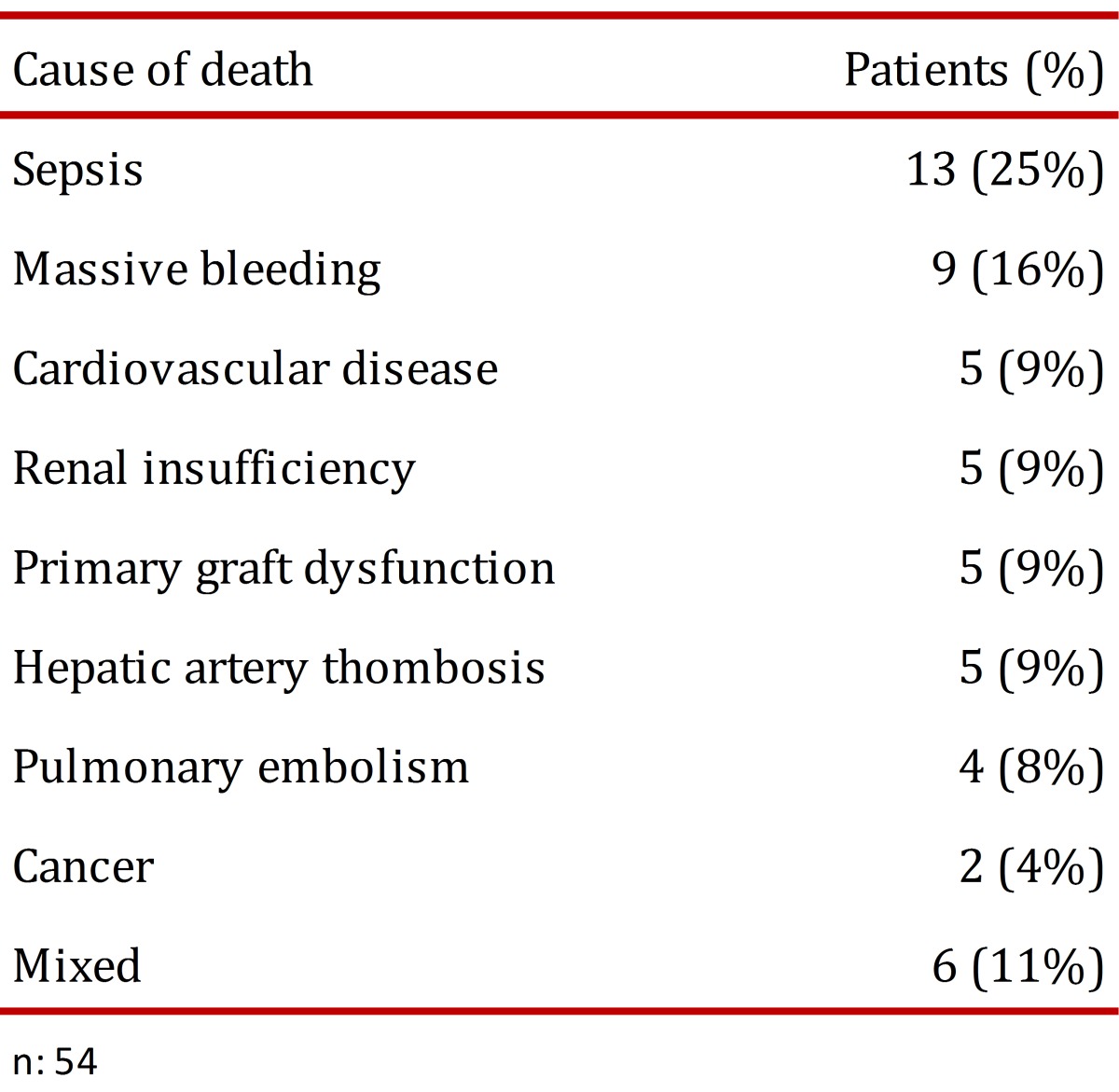
Causes of death for liver transplant patients, Pablo Tobón Uribe Hospital, Medellín, Colombia.

### Perioperative complications

Postoperative bleeding was observed in 22% of the patients, and 20% required reoperation. Reperfusion syndrome was confirmed in 2% of the patients. It was the cause of death for one individual. Primary graft dysfunction was observed in 11 patients (4.5%).

### Vascular complications

Complications in the hepatic artery were observed in 13 patients (5%). Early thrombosis (occurring within the first 30 days) was the main complication (3.7%) for 5 patients with multiple organ failure leading to death. Hepatic artery stenosis was observed in 4 patients. It appeared at late stages and required percutaneous endovascular management. Portal vein complications were observed in 7 patients (3%), five of whom had early portal vein thromboses. One patient had late-stage hemodynamically significant portal vein stenosis. Complications were found either in the hepatic veins or in the venous outflow tract of 3% of the patients. Stenosis was more frequent than thrombosis. Likewise, one of the patients required reoperation since the stenosis occurred at a very early stage; the other patients were successfully treated by the endovascular route. 

### Biliary complications

A total of 35 patients (14.5%) had biliary complications. Of all the injuries, 10% were due to anastomotic stenosis of the biliary tract. In 60% of these cases, the injuries occurred early (within the first 30 days). These patients received endoscopic treatment with dilatation and implantation of biliary prostheses; this procedure was successful in more than 80% of the cases, and only some patients required surgical reconstruction. Non-anastomotic stenosis of the biliary tract occurred at late stages in 2% of the patients. Bile leaks were found in 3% of the patients. In almost all of the cases of bile leaks, they were concomitant with injuries due to stenosis. Of all the cases with biliary complications, 50% were associated with infectious processes originating in the biliary tract and the abdominal cavity. 

### Infectious complications

Ninety-eight patients (41%) had some kind of infection. In 80% of these cases, they occurred within the first 30 days. Bacterial Infections were observed in eighty-four patients (38%). In 50% of these cases, infections originated in the abdomen and included surgical wound infection, cholangitis, and peritonitis. Other foci of infection, in order of frequency, were: lungs, urinary tract, primary bacteremia, and soft tissue infections. As for viral infections, CMV infection was observed in 10% of the patients. Likewise, 80% of these patients presented with this infection after the second month. Herpes zoster was found in 4% of the patients, and herpes simplex in 3%. These infections occurred at late stages, except for one case. Invasive fungal infections were observed in 6 patients (2.5%). The most common germ was *Candida* non-albicans, whose infection originated in the urinary tract in 50% of patients with this infection. Two patients were diagnosed with *Aspergillus *infection. One of them had pulmonary disease with early diagnosis and well-timed treatment, thus yielding a favorable outcome. The other patient, however, had a disseminated disease with infectious endocarditis and late diagnosis. The outcome was death, despite surgical management and treatment with voriconazole and echinocandin. Two patients (0.8%) were found to have late-stage tuberculosis: one with lymph node tuberculosis and the other with tuberculous meningitis. Both were diagnosed during the first weeks of symptom onset and the treatment was well-timed and successful; thus no patients died.

### Other complications

Acute rejection was confirmed through biopsy in 73 patients (30%). Additionally, 80% of these acute rejections occurred within the first three months, and were classified as mild to moderate in the biopsy; thus, they had no significant impact on patient or graft survival. Chronic rejection, in turn, occurred in 7 patients (3%), and was preceded by acute rejection in 85% of these patients. Also, 30% of such patients lost the graft despite the change in immunosuppressant medication. Liver retransplantation was performed in 6.6% of the patients. The main causes were ischemic cholangiopathy, chronic rejection, and hepatic artery thrombosis. In addition, 25% of the patients developed renal failure. Less than 10% of these patients required renal replacement therapy. Arterial hypertension was observed in 

27% of the patients, dyslipidemia in 27%, diabetes mellitus in 8%, and obesity in 7%. Recurrent HCV infection was present in all patients who were transplanted with positive viral load, although clinically significant chronic hepatitis was present in 30% of patients, some received antiviral therapy, one patient underwent liver retransplantation and other two died from the disease. There was no single case of fibrosing cholestatic hepatitis. As for the appearance of de novo neoplasias, they were documented in 8 patients (3%), excluding malignant skin lesions. Eight percent of the patients with hepatocellular carcinoma experienced relapses. However, only one of them had a severe condition, metastasis, and death due to neoplasia.

As to the quality of life of patients undergoing liver transplantation there was no formal assessment, however we documented that patients without serious complications returned more quickly (after 3 months) to their normal daily activities, led a normal family life including the possibility of parenthood and returned to work.

## Discussion 

The liver transplantation had to undergo a number of modifications in recent years in order to generate the results we see today. For the last 3 decades it has been accepted as a therapeutic option for patients with advanced liver disease 6. Although there are various reports of results obtained in different medical centers around the world, with more than 1,000 patients and over 10 years of follow-up, this is one of the few documents which reports the liver transplantation experiences of a center in Colombia.

In this study, patient and liver graft survival rates after 5 years were72% and 68% respectively. These rates are comparable to those reported in the U.S. and Europe 3,7. This is relevant, considering that our country has economic limitations and less experience in carrying out this kind of procedure.

As for other reports from the region, Buckel *et al*. 8, and Castro *et al*. 9, reported similar survival rates in Chile and Brazil, the latter being the country in the world in which the seventh greatest number of liver transplants are carried out 10. In recent years, several centers have reported patient survival rates of more than 90% after one year. We therefore aim to achieve the same excellent results.

In this study, there were high mortality rates during the first year. The leading causes were sepsis with multiple organ failure and massive bleeding. This situation improved in the later years because the surgical team became more experienced and perioperative care improved. Indeed, the length of stay in the ICU may seem long; however, the early extubation protocol has reduced this time to 2 days in recent years. Likewise, hospital stay has been reduced to 12 days.

Infectious complications were frequent, affecting 40% of the patients. Nevertheless, the rate was lower than those reported in early studies on liver transplantation, in which 60% of the individuals were infected 11. Results were also comparable to those recently obtained by large medical centers 12. Of these complications, bacterial infections were the most common. They appeared especially during the first 2 months and the most common source was the abdomen, followed by the lungs and the urinary tract. This is consistent with international data. Viral infections and invasive fungal infections occurred at a rate similar to the one reported by other studies, despite the fact that the antimicrobial prophylaxis protocol for cytomegalovirus and fungi is only applied in patients with known risk factors 13,14. Mycobacterial infections, in turn, occurred in 0.8% of the patients, an important fact considering their moderate prevalence in Colombia 15. This is consistent with other studies in which infection rates are expected to range from 1 to 6% 16. The clinical presentation was extrapulmonary disease, which is not uncommon in this population and hinders early diagnosis.

In general, vascular complications occurred in 11% of the patients. They consisted of hepatic artery thromboses, which appeared early, and in 3.7% of the patients. This percentage is similar to the one described by Duffy *et al* 17. Hepatic artery thrombosis continues to be one of the most feared complications in liver transplantations as it is associated with death in 33% of patients, and with graft loss in 53% of patient 18. In this study, 50% of the patients with hepatic artery thrombosis died from multiple organ failure, and the remaining 50% required early or late liver retransplantation despite the surgical or endovascular measures taken.

As for biliary tract complications, they occurred in 14.5% of the patients, which is consistent with worldwide statistics: 10 to 25% 19-22. Anastomotic stenosis was the most frequent type of injury in this study. It is related to an increase in infections, procedures, and hospitalizations, thus leading to an increase in costs. Biliary leaks, which are associated with not using the biliary T-tube, occurred in low proportions.

In terms of acute and chronic rejection, the results of this study are similar to those recently reported by Pfitzmann et al. in Germany, who found acute and chronic rejection in 41 and 3.5% of the patients respectively 12. 

Liver cirrhosis due to HCV is the main etiology in many worldwide centers where liver transplant is done, but in our center it was only 12% evidencing the low prevalence of HCV in Colombia. 

Survival rates for patients with hepatocellular carcinoma were similar to those of other transplant patients. Relapse rates were lower than 10%, which is similar to the findings reported in the literature when the Milan criteria are met 23. Regarding long-term complications such as chronic renal failure, diabetes mellitus, arterial hypertension and dyslipidemia, rates were lower than those reported in the literature; this could be explained by the fact that our follow-up period was shorter than that of the studies in other centers around the world. Likewise, long-term follow-up was difficult to maintain for some patients due to a local health system which allows for changes in the sites at which patients are seen.

The alcoholic liver disease was the leading indication for liver transplantation in our series, similar to what´s observed in other Latin American countries, however recurrence rates of alcohol was 16.7% overall and only 5.6% severe recurrence that led to liver graft dysfunction.

There are only 5 active liver transplant centers in Colombia, and two of them are located in Medellín. In our country, liver transplant waitlists are small due to the high rate of organ donations, particularly in our region (32 per million inhabitants), thus the waiting time is short and the mortality of the individuals on the waitlist is very low (less than 5%). This report is a good example for other liver transplant centers in developing countries with limited economic resources, e.g. some centers in Asia and Latin America, where good results may be obtained. The high quality of the donors is worth mentioning, particularly in terms of age and cause of death.

The long-term follow-up of some patients and the limited number of cases can be considered to be the limitations of this study. We chose to analyze only the patients who underwent their first liver transplantation and excluded individuals who underwent retransplants, as well as those with combined liver-kidney transplantation and multivisceral transplantation, since the features of these groups of patients are not comparable.

## Conclusion

Liver transplantation is an effective and proven therapy for acute and chronic liver diseases in selected patients. It is now available in Colombia, and its results in the Pablo Tobon Uribe Hospital in Medellin, Colombia, are comparable to those of other medical centers in the world in terms of complications and medium-term survival.
